# Psycho-social impact of orthogathic sugery

**DOI:** 10.4317/jced.53007

**Published:** 2016-12-01

**Authors:** Paolo Cariati, Rocío Martínez, Ildefonso Martínez-Lara

**Affiliations:** 1 Oral and Maxillofacial surgery resident. Hospital Universitario Virgen de las nieves, Granada, Spain; 2 Social Psychology Department. University of Granada. Campus Cartuja s/n. 18001, Granada, Spain; 3 Maxillofacial surgeon. Hospital Universitario Virgen de las nieves, Granada, Spain

## Abstract

**Background:**

Orthognathic surgery is a branch of maxillofacial surgery. It carries out the treatment of the facial skeleton asymmetries and deformities. The patients who ask for this surgery are often young people who usually refer symptoms related to dental malocclusion, difficulty eating and temporo-mandibular pain. These physical symptoms are often accompanied by psychological symptoms triggered by their physical appearance such as low self-esteem, self-confidence and negativism about their social and emotional future.

**Material and Methods:**

Patients with skeletal malformation of facial bones, consisting in Class II, III, open bite and asymmetries, underwent to orthognathic surgery in our center agreed to participate voluntarily in this study. They answered a questionnaire regarding several psychosocial variables.

**Results:**

Orthognathic surgery helps to improve patient’s psychosocial well-being.

**Conclusions:**

Patients with dentofacial deformitiesexperience physical and psychological, oftentimes underestimated by society. A combination of orthodontic treatment and reconstructive surgery is often a necessity to restore function and psychosocial well-being.

** Key words:**Orthogathic surgery, psychosocial consequences, mood, emotions, sense of power, motivation, satisfaction, social changes, satisfaction.

## Introduction

Orthognathic surgery is used to correct facial deformities affecting the jaw, maxillary and teeth. The aim of ortognathic surgery is to harmonize the facial skeletal structure and it could improve the physical and psychological symptoms of the patients (1). In our opinion, people with facial deformities may be victims of prejudice and discrimination due to his physical appearance. They could appear less employable and efficient than people with normal physical appearance (1). According to Miguel JA *et al.* patients who demand orthognathic surgery frequently present negative psychological symptoms related to their physical appearance (2). Furthermore, it seems clear that depending on the type of jaw deformities we could observe different psychological consequences (3). In fact, a comparative study of skeletal class II and skeletal class III patients showed that skeletal class III patients had stronger feelings of insecurity regarding their facial appearance (4). In addition, as Hunt *et al.* pointed out most of patients who request surgical-orthodontic treatment wish to improve their facial and dental appearance (5). According to these authors, patients expect to improve their self-steem and self-confidence. Moreover, they also look forward to feel less social embarrassment.

In line with the previous ideas, numerous studies showed that the satisfaction level with this surgery is usually high. Indeed, it has been observed an improvement of the physical and psychological symptoms (6). Some of these studies even claim that the progress in terms of personality and physical appearance acceptance could be higher than the improvement of symptoms such as pain, clicking, locking and hipomobility (7). Even though literature shows how patients underwent to orthognathic surgery experienced a quality of life improvement (8), several and relevant psychological and psychosocial variables have been slightly considered. In this regard, the main aim of our paper is to analyze the impact of orthognathic surgery in certain scarcely studied psychological variables such as mood and emotions (anger, sadness, anxiety, hostility), sense of power and control or satisfaction with life. In addition, other variables, as self-esteem, satisfaction with their appearance, satisfaction with surgery, social and family relations changes after surgery were analyzed. Interestedly the impact of the previous physical appearance on the patient’s feelings (social embarrassment, depression, etc.) was examined. Finally, we also evaluated the patient’s motivation to demand orthognathic surgery and the more negative aspects associated with surgery.

## Material and Methods

-Method

•Participants

26 patients (10 males, 16 females) with skeletal malformation of facial bones, consisting in Class II, III, open bite and asymmetries, underwent to orthognathic surgery in our center agreed to participate voluntarily in this study (61.5% women, 38.5 % men). Their mean age was 29.65 years old (SD = 7.07).

•Orthodontic treatment and surgery

All patients completed a specific orthodontic treatment previous to surgery. The surgery planning was performed in close collaboration with patient’s orthodontist. All patients in the study were underwent to bimaxillary osteotomies. Planning surgery was performed employing the traditional technique with classical model surgery and splint or with virtual planning using Simplant O & O program. The average length of postoperative hospital stay was 2 days. An elastic occlusal locking was maintained for 2 week after surgery. We ensured that patients filled the questionnaire at least six month later of the surgery intervention.

-Materials

•The questionnaire contained the following measures:

•Scale for Mood Assessment (EVEA). Participants were asked to indicate the degree to which they felt 16 different emotional responses when thinking about the achieved through the surgery. Specifically the EVEA is composed by the following subscales (9) (Sanz, 2001): anxiety (“nervous”, “tense”, “anxious”, and “restless”), anger-hostility (“irritated”, “angry”, “annoyed”, and “displeased”), sadness-depression (“melancholy”, “depressed”, “downcast” and “sad”), and happiness (“happy”, “optimistic”, “joyful”, and “cheerful”). Participants indicated the extent to which each emotion described their feelings on a scale ranging from 1 (“Not at all”) to 10 (“Completely”). The internal consistency reliability was appropriate for the subscales of anxiety (α =.72) anger-hostility (α =.51), sadness-depression (α =.68) and happiness (α =.67). It is important to note that the item “melanchology” was removed from the sadness-depression subscale due to low reliability. The mean score for each subscale emotions was calculated.

•Sense of Power and Control was measured using two items to capture the extent to which the participant felt powerful / power-less and with control over the situation when thinking about the physical appearance achieved through the surgery. The sense of power is an important “psychological state related with the perception of one’s capacity to influence others” (Galinsky, Gruenfeld, & Magee, 2003; p.314) (10). In order to measure it participants expressed their opinion on a scale ranging from 1 (“Not at all”) to 10 (“Completely”).

•Self-Esteem and Personal Satisfaction. Two items were used to capture the extent to which the participant assess the self-esteem and the satisfaction when comparing with the previous self-steem (1= “Very Bad” to 5 = “Very Good”) and satisfaction before the surgery (1= “Very Unsatisfied” to 5 = “Very Satisfied”). We also added two additional items (α = .79) to examine patient’s perception of their actual facial’s appearance comparing with previous facial’s appearance (1= Much worse than before to 7 = Much better than before). Finally, a single item was included to evaluate how their familiars, friends and workmates appraised the physical appearance achieved through the surgery intervention (1= Much worse than before to 7 = Much better than before).

•Satisfaction with Life (Diener, Emmons, Larsen y Griffin, 1985) (11). We used the Spanish version of the Satisfaction with life scale described by Atienza, Pons, Balaguer and Garcia-Merita (2000) (12). This 5- item scale allows researchers to measure global cognitive judgments of one’s life satisfaction. Participants were asked to rate the extent they agree or disagree with each of the 5 items on a Likert scale (1= Strongly disagree; 5 = Strongly agree). We computed an index averaging all the items and emotional reactions (α = .87), which indicates the extent to which participants held satisfaction with their life after the surgery.

•Motivation and Reasons for surgery. Firstly, we asked participants to write down the main reasons (maximum 3) behind the decision of undergoing a surgery. Afterwards, participants were presented with three items that assessed the extent that the decision of undergoing a surgery was motivated by health concerns (e.g. Difficulties in speaking and swallowing) and to what extent was driven by social and personal motives (e.g. to make oneself feel better or to be accepted and integrated into the society). Scores of the social and personal two items were averaged (α =.66).

•Social, Family and Professional relationships. Three items measured changes in social, family and professional relationships after the surgery. We modified the scale of response used by Almeida Silveira Carvalho, de Santana Santos, Studart Rocha, Amorim Gomes, Dias de Oliveira e Silva (2013) in order to obtain a more accurate measure. So that, instead of using a 3 point scale (Good, fair or bad) we inserted a Likert scale of 5 points (1= Much worse than before, 2 = A bit worse than before, 3 = Same than before, 4= A bit better than before, 5 = Much better than before).

•Impact of the previous physical appearance on the patient’s feelings. Importantly, we measured how physical appearance made patients feeling in their lives. Specifically, we added two items to evaluate the extent participants felt “embarrassment” and “shame” (α =.78). In addition, we also included six items (α =.91) to study the extent that patients had “difficulties in concentrating, sleeping or relaxing” or the extent they felt “upset or irritable” when they thought about their physical appearance.Lastly, a single item was included to evaluate the extent that participants felt depressed due to their appearance.

•Aspects related with the Surgery. The questionnaire also included some items to examine if: a) patients got the expected results, b) patients recommend surgery to their family members and c) what were the most bothersome aspect of surgery (Alves e Silva, 2013). In addition, we added two items to analyze f) to what extent the surgery has solved the problem that patient had and g) to what extent patients feel positive and self-confidence after the surgery (1 = Not at all to 5 = A lot).

Finally, participants reporting socio-demographic information: age, sex and nationality. After completing the questionnaire, participants were thanked for their participation.

## Results

-Statistical analyses

We conducted several t-test comparisons with the midpoint of each scale in order to determine the level of the different variables included in our study. We also carried several t-test paired comparisons in order to compare the differences between some varia-bles in our study (SPSS for Windows, version 17.0).

-Scale for Mood Assessment (EVEA). In order to examine the mood of patients when thinking about the physical appearance achieved through the surgery, a comparison with the midpoint of the scale was conducted. Results showed that patients presented a low level of negative emotions: anxiety (M =.66, SD= 1.29; t(25) = 17.13, *p* < .01), anger-hostility (M =.18, SD= .55; t(25) = 44.43, *p* < .01) and sadness-depression (M =.32, SD=1.09; t(25) = 21.66, *p* < .01). However, they informed to feel a greater level of happiness (M =8.48, SD= 1.43; t(25) = 12.40, *p* < .01).

-Sense of Power and Control. A comparison with the midpoint of the scale showed that patients presented a high level of sense of power (M = 7.16, SD= 2.68; t(24) = 4.01, *p* < .01) and a high level of sense of control after the surgery (M = 8.31 SD= 2.25; t(25) = 7.46, *p* < .01).

-Self-Esteem and Personal Satisfaction. Results showed that self-steem (M =4.54, SD= .50; t(25) = 20.44, *p* < .01) and personal satisfaction (M =4.92, SD=.27; t(25) =45.46, *p* < .01) after de surgery was notably high. Similarly, we found that patients (M =6.63, SD= .48; t(25) = 33.24, *p* < .01) and their familiars and friends (M = 6.42, SD= .64; t(25) = 23.16, *p* < .01) perceived them much better than before the surgery.

-Satisfaction with Life. The analyses conducted showed that the satisfaction with life of the patients evaluated was significantly high (M = 4.1, SD= .68; t(25) = 11.92, *p* < .01).

-Motivation and Reasons for surgery. Results showed that both healthy (M = 5.73, SD= 1.77; t(25) = 6.39, *p* < .01) and social and personal (M = 4.63, SD= 1.72; t(25) = 3.35, *p* < .01) motives influenced importantly the decision of undergoing a surgery. In addition, when we examined the reasons described by patients in the questionnaire, we observed that 50% of the motives were classified as personal and social (e.g. “To be complexes”, “Social Exclusion” or “To improve the psychical appearance) whereas a 45.71% were motives related with their health (e.g. “Jaw Pain” or “Difficulty chewing”). The 4.28 % of reasons described by the patients were motives not included in the previous categories (e.g. “trust on the surgeon” or “Recommendation of the dentist”).

-Social, Family and Professional relationships. Significant differences were found when comparing with the midpoint of the scale showing that social (M = 3.76, SD= .92; t(24) = 6.80, *p* < .01), family (M = 3.28, SD= .67; t(24) = 5.75, *p* < .01) and professional relationships (M =3.72, SD= .89; t(24) = 6.84, *p* < .01) improved after the surgery.

-Impact of the previous physical appearance on the patient’s feelings. Interestingly, results showed that patients felt quite embarrassed and ashamed (M = 4.21, SD= 1.86; t(25) = 1.94, *p* < .01) and also they informed to feel depressed (M =4.50, SD= 2.23; t(25) = 2.28, *p* < .01) throughout their lives before being operated. Although, in this case, a comparison with the midpoint of the scale did not show significant differences, we also found that they felt moderately irritable and uncomfortable (M =3.29, SD= 1.72; t(25) =.60, *p* > .05) due to their physical appearance.

**Figure 1:**
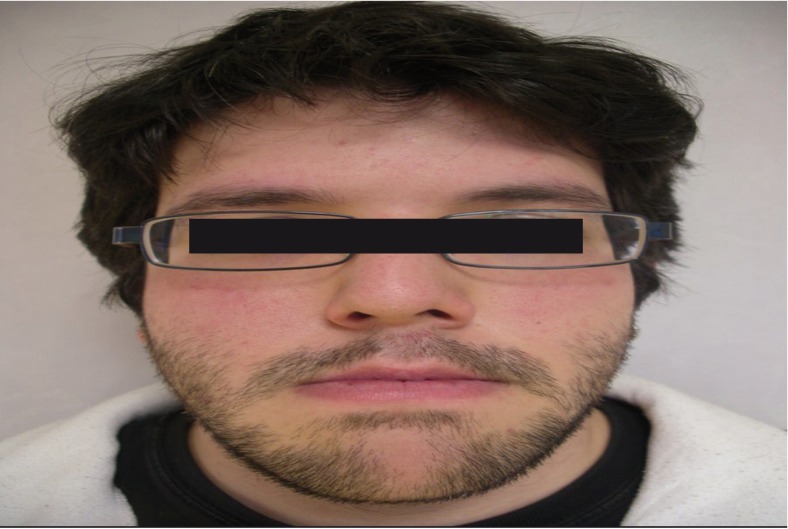
Patient’s appearance before surgery.

**Figure 2:**
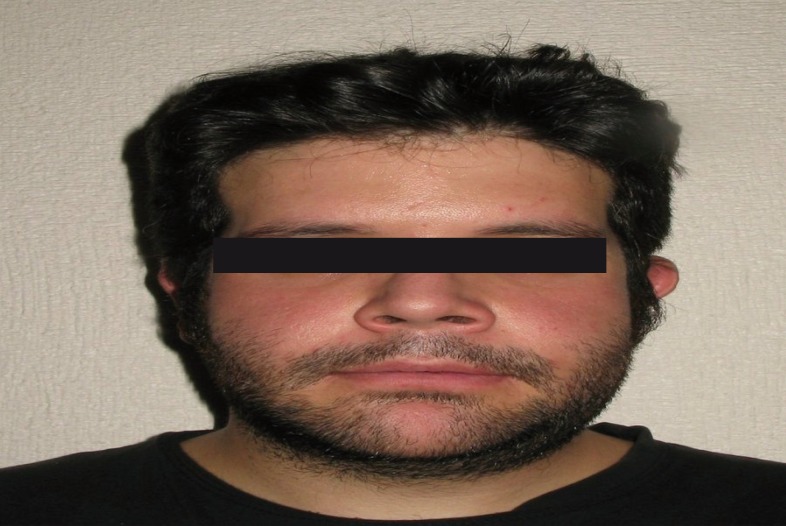
Patient’s appearance after surgery.

**Figure 3:**
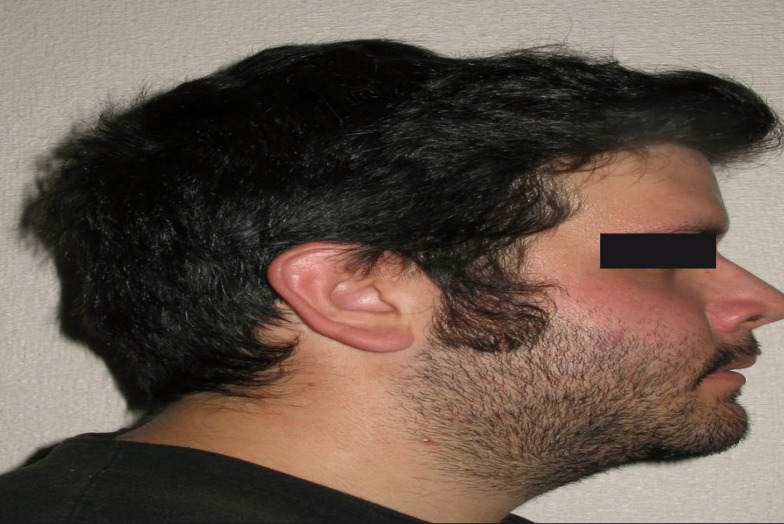
Patient profile after surgery.

-Aspects related with the Surgery. 96% of patients informed to get the results and to recommend surgery to their family members. Results also showed that the most bothersome aspects of the surgery were related with: eating issues (28.6%), the first week post-surgical (23.8%), paresthesia (21.4%), 24 hour postsurgical (9.5%), the immediate postoperative phase (7.1%), hospitalization (4.8%), immobilization (2.4%) and other motives non specified (2.4%). Finally, the analyses allowed us to find out that patients highly considered that the surgery solved the problem they had (M =4.50, SD= .70; t(25) = 14.42, *p* < .01). Moreover, we also observed that they feel positive and self-confidence after the surgery (M =4.42, SD= .64; t(24) = 15.24, *p* < .01), (Figs. 1-3).

## Discussion

Orthognathic patients present an objectified dentofacial deformities and, consequently, physical and psychological symptoms due to their pathology. So that, according to Rustemeyer *et al.* function, aesthetics, and even psychological aspects should be considered equally when planning surgery (13). Regarding the psychological aspects, the present research focused on important variables related to orthognathic surgery. Specifically, we analyzed the impact of the surgery on: a) mood and emotions (anger, sadness, anxiety, hostility) b) sense of power and control, c) self-esteem and personal satisfaction, d) satisfaction of life, e) motivation and reasons for surgery, social, family and professional relationships changes, f) impact of the previous physical appearance on the patient’s feelings and finally we also explored g) other issues related with the surgery ( e.g. what were the most bothersome aspect of surgery).

Results of this research demonstrated that orthognatic sugery impact in a very positive way on the patient’s mood. Specifically we found that when patients thought about their physical appearance achieved through the surgery, patients presented a low level of negative emotions: anxiety, anger-hostility and sadness-depression). In contrast, they reported to feel a greater level of happiness. Furthermore, our findings also showed that patients also felt with a high sense of control and power over their lives. According to Galinsky *et al.* (10) (2003) power is a psychological state observed when people perceive themselves as able to influence others. So that, we might conclude that the psychical appearance achieved through the orthognathic surgery makes patients feel as more influential and powerful. In this line of reasoning, our research also confirmed that orthognathic influenced on the self-steem and the personal satisfaction that patients had after the surgery. They informed not only that they perceived themselves much better than before the surgery but also that their relatives and friends thought the same. In our opinion, these results underline the importance of orthognathic surgery concerning the functional aspects but importantly regarding the psychological aspects.

The present research was able to make progress in the study on the consequences of orthognathic sugery. Interestedly, patients’s satisfaction with life was also notably high. We are convinced that this is a very interesting result due to the importance of this psychological variable. Life satisfaction is “a global assessment of a person’s quality of life according to his chosen criteria” (14) (Shin and Johnson, 1978; p. 478). We would like to stress how their psysical appeareance after the surgery impacted on the evaluation that patients make about the quality of their lives. Our findings showed that the cognitive judgments that patients made about their lives after the surgery were highly positive. It is therefore not surprising that patients also informed that social, family and professional relationships improved after the surgery. In other words said, surgery impacted not only on the psychological and healthy aspects of the patients but also on their social context.

In addition, our study was also aimed to examine the patient’s motivation for surgery. The findings showed that both healthy and social and personal motives influenced importantly the decision of undergoing a surgery. It seems clear that patients were motivated not only because of they experiment problems related to dental malocclusion, difficulty eating and temporo-mandibular pain, but also because these physical symptoms were often accompanied by psychological symptoms triggered by their physical appearance. In this regard, our research also highlight how patients informed to feel quite embarrassed, ashamed and depressed throughout their lives before being operated. We also found they felt moderately irritable and uncomfortable due to their physical appearance. So that, we claim the importance of this type of surgery since their appearance causes double suffering because it affects the psychological and the psychical sides of their lives. Importantly, most of them informed to get the results that they expected and that the surgery solved the problem they had.

Concluding, we would to stress that this report contains two points that are central to us: firstly, our analysis indicated that orthognathic surgery could improve physical and psychological symptom of patients with dentofacial deformities. Thus, the common knowledge that patients demand surgery only for aesthetic reasons should be reconsidered. Patients affected by dentofacial deformities should not be compared with patients with psychiatric disorders such as dysmorphophobia. In fact, several reports showed that patient with dentofacial deformity present greater rates of satisfaction than cosmetic surgery patient (15). In our point of view, the reason of these results could be that most of the people demanding cosmetic surgery presenting normal physiognomy, although they are dissatisfied with their appearance. However, patient requiring orthognathic surgery presenting an objectified dentofacial deformities and, consequently, physical and psychological symptoms due to their pathology. In fact, several reports highlight that patients underwent to orthognathic surgery experimented a great improvement in physical and psychological problems. So that, in our point of view, orthognathic surgery should be considered as important as other kinds of reconstructive and functional surgeries and not only an aesthetic procedure.

Secondly, professionals engaged in orthognathic surgery know the calvary that orthognathic patients live due to the length of orthodontic treatment and immediate postoperative period morbidity. To all this must be added thephysical and psychological suffering caused by dentofacial deformities, oftentimes underestimated by society. Fortunately, patient efforts are rewarded with brilliant results in terms of physical and psychological improvement. In fact, as Bellucci *et al.* underline in their work, a combination of orthodontic treatment and reconstructive surgery is often a necessity to restore function ( in terms of oral-motor development, airway, and speech) and psychological well-being ( in terms of social interaction and quality of life) (16). With these ideas in mind, we would to emphasize that orthognathic surgery should not be considered a luxury surgey. Indeed we strongly believe that patient affected by dentofacial deformities need this procedure to achieve their physical, psychological and functional well-being, and we are firmly convinced that public health institutions should respond to this need.
